# An n-type semiconducting diazaporphyrin-based hydrogen-bonded organic framework†

**DOI:** 10.1039/d4sc03455d

**Published:** 2024-07-11

**Authors:** Takahiro Sakurai, Tappei Tanabe, Hiroaki Iguchi, Zhuowei Li, Wakana Matsuda, Yusuke Tsutsui, Shu Seki, Ryotaro Matsuda, Hiroshi Shinokubo

**Affiliations:** a Department of Molecular and Macromolecular Chemistry, Graduate School of Engineering, Integrated Research Consortium on Chemical Science (IRCCS), Nagoya University Furo-cho, Chikusa-ku Nagoya 464-8603 Japan hshino@chembio.nagoya-u.ac.jp; b Department of Material Chemistry, Graduate School of Engineering, Integrated Research Consortium on Chemical Science (IRCCS), Nagoya University Furo-cho, Chikusa-ku Nagoya 464-8603 Japan ryotaro.matsuda@chembio.nagoya-u.ac.jp; c Department of Molecular Engineering, Graduate School of Engineering, Kyoto University Nishikyo-ku Kyoto 615-8510 Japan seki@moleng.kyoto-u.ac.jp

## Abstract

Significant effort has been devoted to the development of materials that combine high electrical conductivity and permanent porosity. This paper discloses a diazaporphyrin-based hydrogen-bonded organic framework (HOF) with porosity and n-type semiconductivity. A 5,15-diazaporphyrin Ni(ii) complex with carboxyphenyl groups at the *meso* positions afforded a HOF due to hydrogen-bonding interactions between the carboxy groups and *meso*-nitrogen atoms. The thermal and chemical stabilities of the HOF were examined using powder X-ray diffraction analysis, and the charge-carrier mobility was determined to be 2.0 × 10^−7^ m^2^ V^−1^ s^−1^ using the flash-photolysis time-resolved microwave conductivity (FP-TRMC) method. An analogous diazaporphyrin, which does not form a HOF, exhibited mobility that was 20 times lower. The results presented herein highlight the crucial role of hydrogen-bonding networks in achieving conductive pathways that can tolerate thermal perturbation.

## Abstract 2

A Ni(ii) diazaporphyrin with carboxy groups afforded a stable HOF through intermolecular hydrogen-bonding interactions, which exhibited high semiconductivity as well as stability toward heating and various solvents.

## Graphical abstract

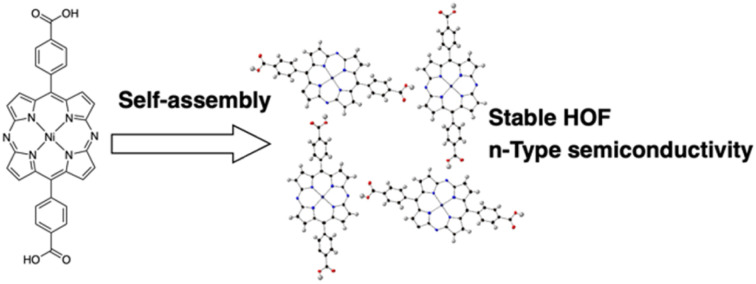

## Introduction

Porous crystalline materials (PCMs) such as metal–organic frameworks (MOFs)^1^ and covalent organic frameworks (COFs)^2^ have been widely studied for their applications in gas storage,^3^ separation,^4^ catalysis,^5^ and drug delivery.^6^ Recently, PCMs that exhibit electronic conductivity through the use of π-conjugated molecules as electroactive components have been extensively investigated.^7,8^ To achieve efficient charge transport, sufficient orbital overlap among π-conjugated motifs is essential to provide conduction pathways for the charge carriers.

Hydrogen-bonded organic frameworks (HOFs) are a class of PCMs constructed by hydrogen bonds,^9^ and are superior to other porous materials in terms of processability and recyclability. However, the chemical and thermal stability of HOFs is generally low due to the weakness of hydrogen-bonding interactions. One effective design strategy to obtain more robust HOFs is to employ rigid and planar π-systems, thus exploiting both π–π interactions and hydrogen bonding.^10^ In such systems, the π-conjugated molecules are stacked in one dimension, thus affording conductive HOFs.^11^

Porphyrins are prospective HOF building blocks due to their rigid and planar structure,^12^ redox activity^13^ and versatile peripheral functionalization.^14^ Various porphyrin-based HOFs that combine permanent porosity with gas-adsorption properties, catalytic activity, and proton conductivity have already been reported.^15^ However, despite reports of porphyrin-based MOFs and COFs that exhibit conductivity/photoconductivity,^16^ the electronic mobility of porphyrin-based HOFs has yet to be studied.

**Fig. 1:**
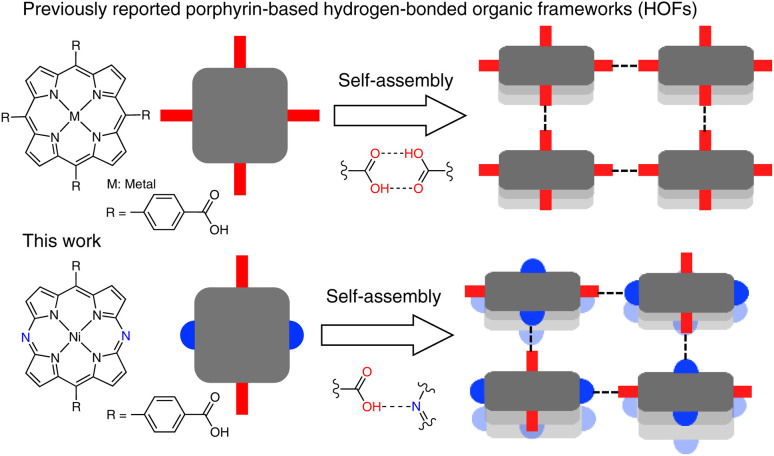
Schematic representation of porphyrin-(previous work; top) and diazaporphyrin-based HOFs (this work; bottom).

In this work, we disclose that a 5,15-diazaporphyrin Ni(ii) complex functionalized with carboxyphenyl groups at the *meso* positions affords a stable HOF with porosity. In this HOF, the *meso*-nitrogen atoms act as intrinsic hydrogen-bonding acceptor sites (Fig. 1). In contrast, conventional porphyrin-based HOFs are assembled through hydrogen-bonding interactions between carboxylic-acid pairs. This feature of the diazaporphyrin-based HOF enables closer proximity of the porphyrinoid skeletons in the resulting HOFs, which would be beneficial for enhancing intermolecular interactions.

The charge-carrier mobility of the HOF was investigated. Flash-photolysis time-resolved microwave conductivity (FP-TRMC) analysis revealed that the diazaporphyrin-based HOF exhibits superior n-type mobility to an analogous diphenyldiazaporphyrin without intercolumnar hydrogen-bonding interactions. To date, n-type conducting PCMs have been limited to quinone-, naphthalene diimide-, and phthalocyanine-based PCMs, while most π-conjugated compounds exhibit p-type conductivity because their electron-donating nature stabilizes radical cation species as the charge carrier.^7,8^ The present diazaporphyrin-based HOF exhibits electron mobility enabled by the high electronegativity of the incorporated sp^2^-nitrogen atoms. Furthermore, the present results highlight the importance of the hydrogen-bonding network in achieving stable electronic conduction pathways in organic materials.

## Results and discussion

### Synthesis and characterization

**Scheme 1:**
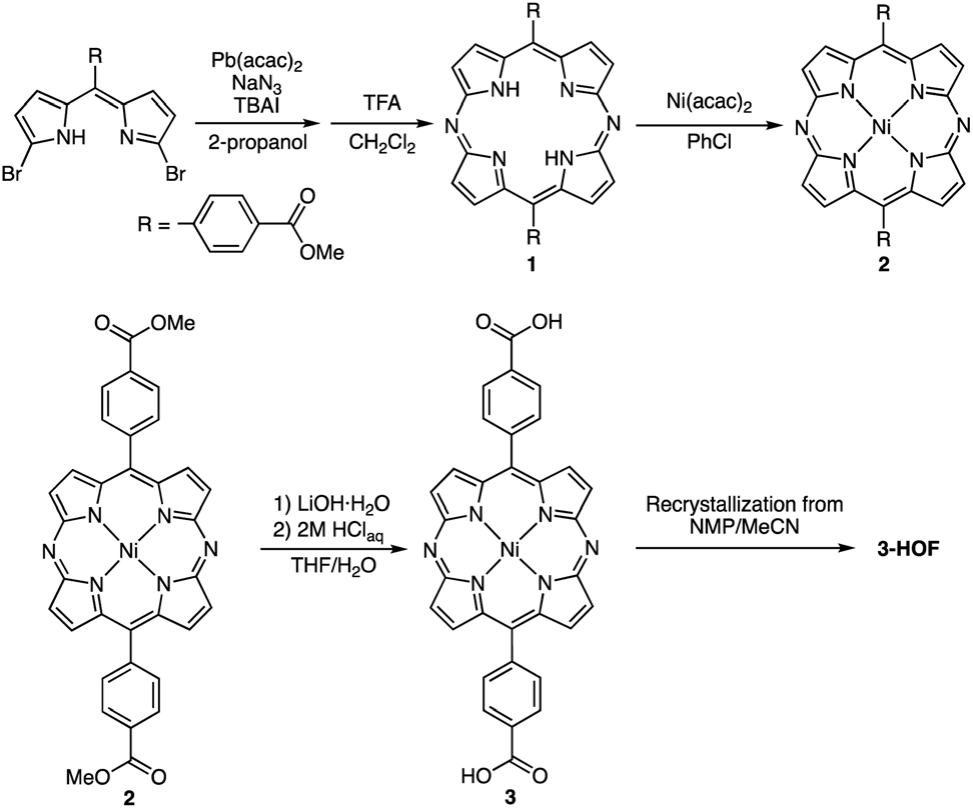
Synthesis of 3-HOF.

Scheme 1 shows the synthetic procedure that leads to the diazaporphyrin-based HOF (3-HOF). Pb-templated cyclization of meso-carboxymethyl phenyldibromodipyrrin with sodium azide afforded the corresponding diazaporphyrin 1 in 16% yield.^17^ Complexation of 1 with nickel acetylacetonate produced Ni(ii) complex 2 in 86% yield. Hydrolysis of 2 under basic conditions quantitatively afforded 3. Finally, recrystallization of 3 from *N*-methylpyrrolidone (NMP)/acetonitrile furnished 3-HOF.

**Fig. 2:**
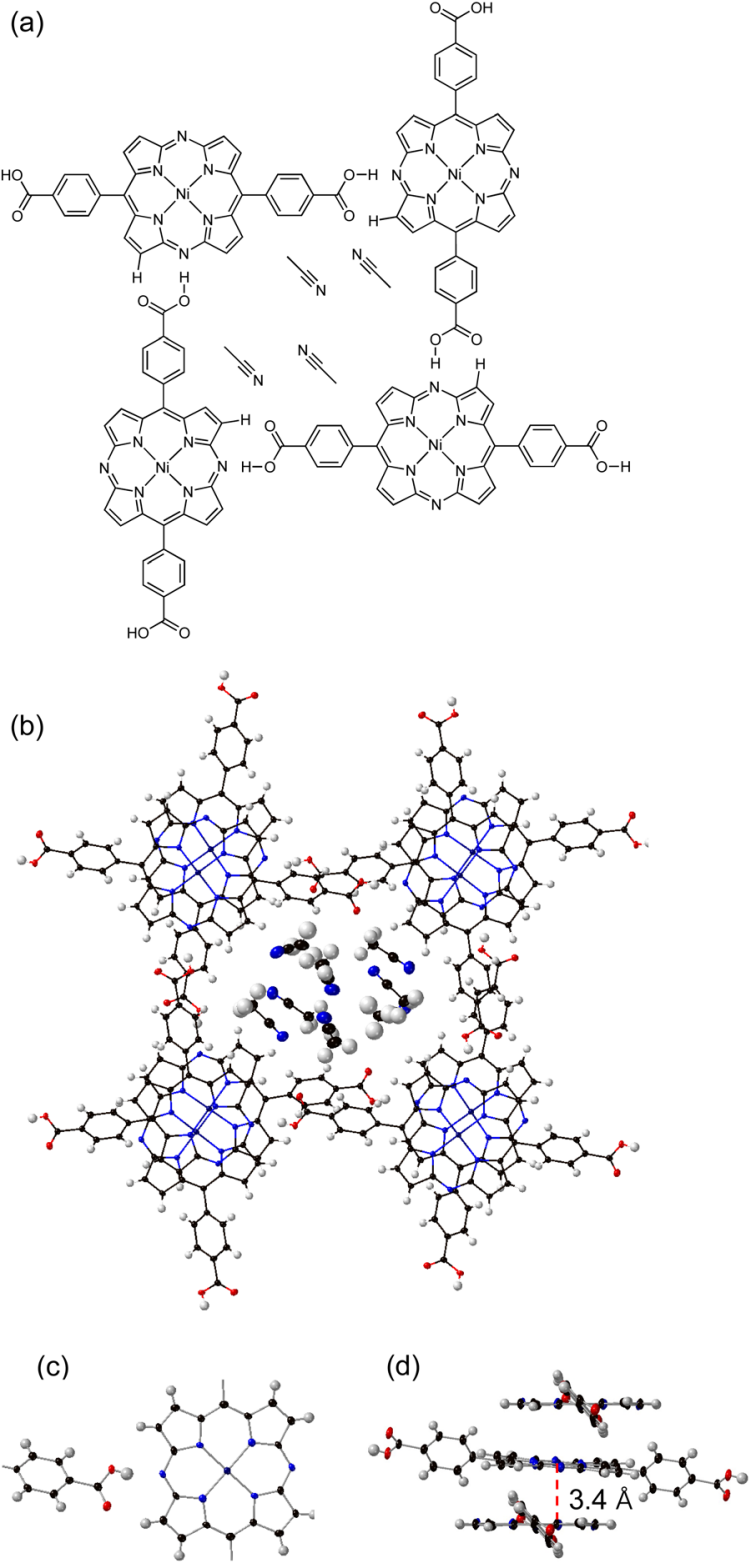
(a) Schematic illustration of the crystal structure of 3-HOF. (b) Top view of the X-ray crystal structure of 3-HOF. (c) Double hydrogen-bonding interactions between the diazaporphyrin cores and the carboxyphenyl groups. (d) Packing structure of three stacked molecules of 3.

A single-crystal X-ray diffraction analysis of 3-HOF unambiguously revealed a 2-D sheet structure formed by hydrogen-bonding interactions between the carboxyl groups and the diazaporphyrin cores (Fig. 2). The FT-IR spectrum of 3-HOF exhibited a broad peak around 2500 cm^−1^ (Fig. S10†), also confirming the presence of intermolecular hydrogen-bonding interactions. The OH groups form hydrogen bonds with the nitrogen atoms at the *meso*-positions. Furthermore, the C=O groups also interact with one of the β-protons. This hydrogen-bonding network among the diazaporphyrin units assembles them into a 2-D sheet structure with rectangular pores, and the 2-D sheets undergo π-stacking to form a porous crystal structure with 1-D channels. The 1-D channels include molecules of acetonitrile, which was used as an anti-solvent for recrystallization. The π-stacked diazaporphyrin pairs were not perfectly parallel. Defining the π–π distance as the mean distance of the four nitrogen atoms from the mean plane of the four nitrogen atoms of the diazaporphyrin skeleton, the measured π–π distance was 3.4 Å.

### Porosity and stability of HOF

**Fig. 3:**
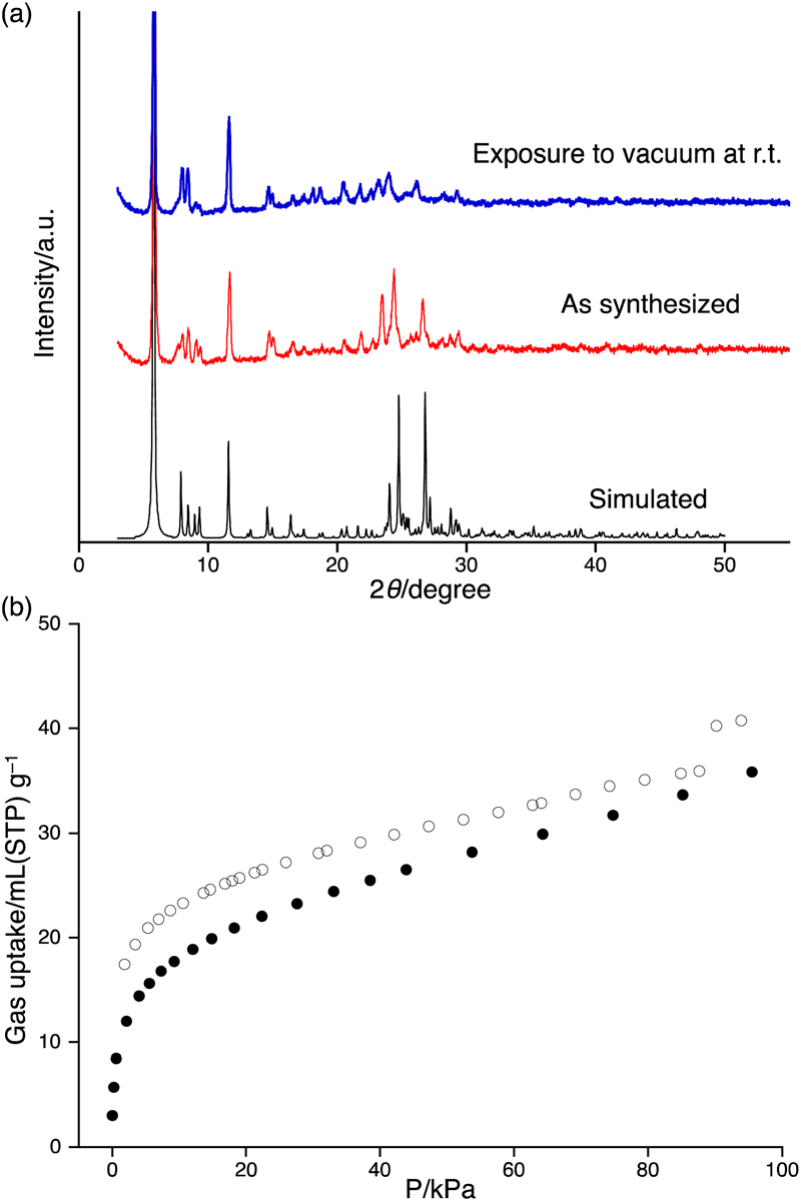
(a) PXRD patterns of the as-synthesized sample (red) and the sample exposed to vacuum at r.t. (blue), together with the simulated PXRD pattern (black) of 3-HOF. (b) Adsorption (●)/desorption (○) isotherms for CO_2_ at 195 K.

We next investigated the stability of the pore structure of 3-HOF. The powder X-ray diffraction (PXRD) peaks agreed well with the predicted diffraction patterns from the single-crystal structure, indicating the absence of polymorphism under the applied crystallization conditions (Fig. 3a). The diffraction pattern of a sample that was activated under vacuum at room temperature exhibited no changes from that of the as-synthesized sample. The existence of pores was confirmed by CO_2_ isotherms, which exhibited type-I characteristics (Fig. 3b).^18^ Furthermore, we confirmed that the solvent molecules in the pores could be eliminated by simply allowing the solid sample to stand under ambient conditions for 12 h.

We then tested the chemical stability of 3-HOF by soaking the crystals in various solvents for 12 h (Fig. S12a†). The HOF structure was stable against the tested solvents, albeit that the diffraction pattern broadened slightly after exposure to THF, probably due to partial dissolution of the crystals in THF. Next, the thermal stability was examined. A thermogravimetric (TG) analysis revealed that the ligand molecule is stable up to 350 °C (Fig. S13†). Temperature-dependent PXRD measurements indicated that the HOF structure gradually collapses at temperatures above 150 °C (Fig. S12b†). We concluded that 3-HOF was not as stable as HOFs that feature hydrogen-bonding interactions between two COOH pairs.^10,15*c*^

### Electrochemistry

The electrochemical properties of 2, 3 and the analogous diphenyldiazaporphyrin Ni(ii) complex 4 were examined using cyclic voltammetry (CV) in order to evaluate their electron-accepting ability (Fig. S14†). The first reduction potentials of these compounds were recorded around −1.2 V. These values are substantially higher than that of Ni(ii) dimesitylporphyrin (−1.77 V),^17^ suggesting that the diazaporphyrin core shows enhanced electron-accepting ability due to the incorporated sp^2^-nitrogen atoms.

**Fig. 4:**
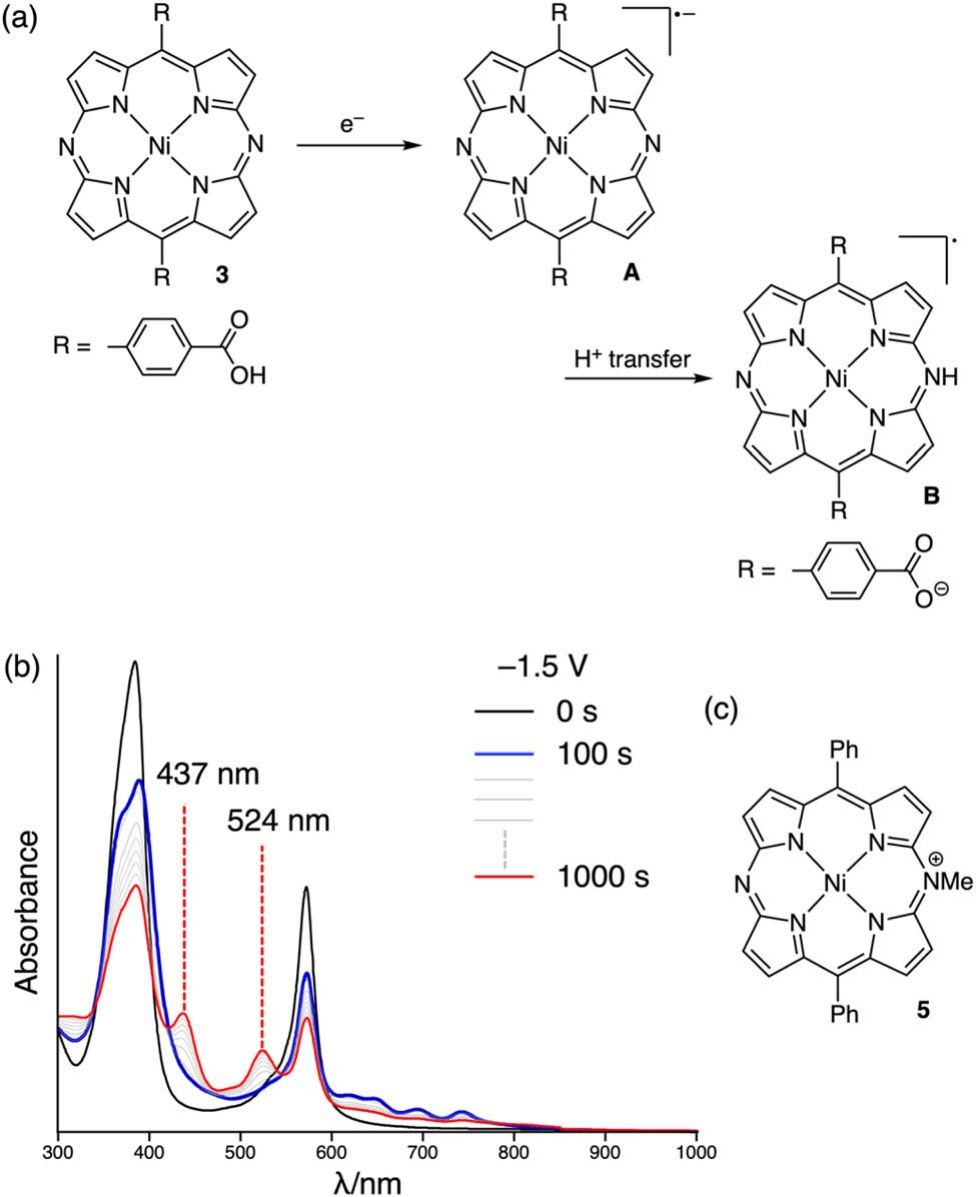
(a) Schematic illustration of the one-electron reduction of 3 involving proton transfer. (b) Change in the absorption spectrum of 3 in DMF upon one-electron reduction. (c) Structure of *N*-methyldiazaporphyrinium cation 5.

Spectroelectrochemical measurements of 3 in solution were also conducted in order to examine a possible proton transfer upon electrochemical reduction (Fig. 4a). One-electron reduction of 3 at −1.5 V induced two-step spectral changes (Fig. 4b). Initially, broad absorption bands appeared between 600–800 nm. The spectrum observed at 100 s is consistent with that of the one-electron reduced species of diphenyldiazaporphyrin Ni(ii) 4, indicating the formation of radical anion A (Fig. S16b†). Subsequently, the emergence of new absorption bands at 437 nm and 524 nm was observed, together with a decreased intensity of the longer-wavelength absorption bands. The final spectrum at 1000 s resembles the absorption spectrum of the one-electron reduced species of *N*-methyldiazaporphyrinium cation 5^19^ (Fig. S16c†). Consequently, we concluded that protonation of radical anion A provides neutral radical B.

### Charge mobility

**Fig. 5:**
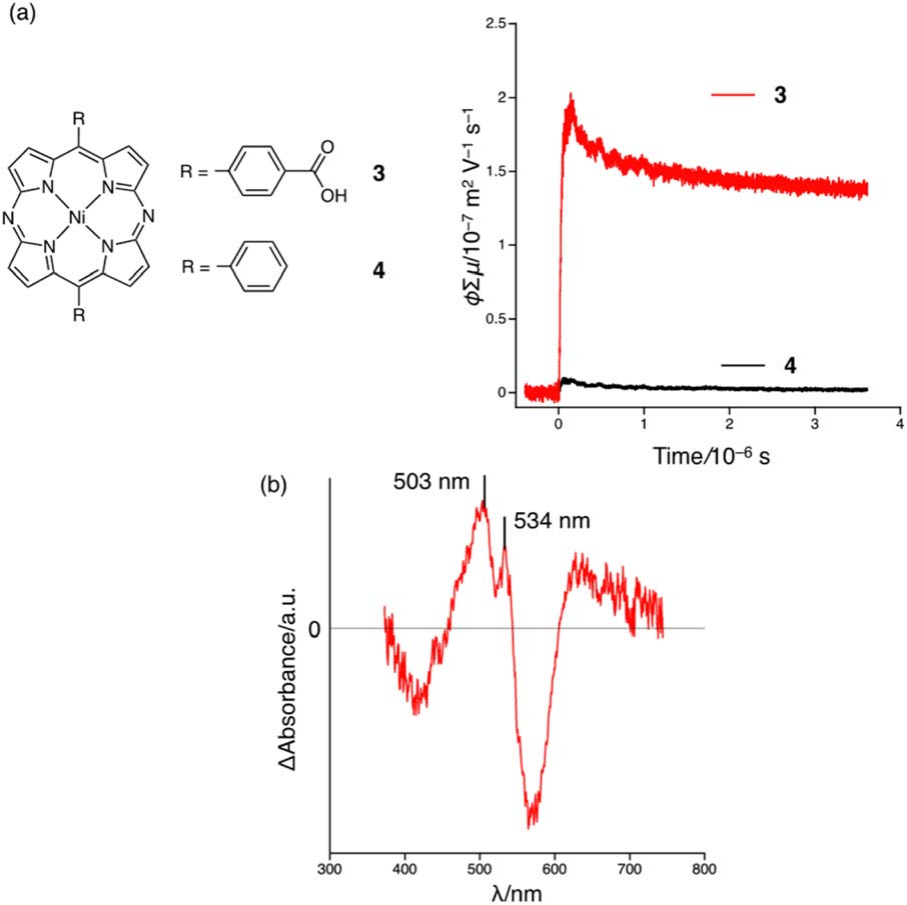
(a) FP-TRMC profiles for as-synthesized 3-HOF (red) and 4 (black). (b) Transient absorption spectrum of 3-HOF in a PMMA film recorded at 0–10 ms after pulse excitation at 355 nm (6.4 × 10^15^ photons per cm^2^).

Then, the charge mobility of as-synthesized 3-HOF was evaluated. FP-TRMC measurement was conducted to examine the intrinsic short-range charge mobility (Fig. 5a). The photocarriers were generated by photoirradiation at 355 nm. A maximum *φ*Σ*μ* (*φ*: carrier generation efficiency; Σ*μ*: sum of electron and hole mobilities) value of 2.0 × 10^−7^ m^2^ V^−1^ s^−1^ was recorded for 3-HOF; this is the highest *φ*Σ*μ* value observed to date for porphyrin-based materials^16^ and MOFs/COFs with embedded conjugated core molecules (Table S2†).^20^ The *φ* value could not be determined because we failed to obtain thin films of 3-HOF. To determine the charge carrier, we measured the transient absorption spectrum of 3-HOF in a PMMA matrix, which exhibited peaks at 503 nm and 534 nm (Fig. 5b). The spectral features resemble the electrochemical absorption spectrum of 3 at 1000 s (red trace) rather than that at 100 s (blue trace) (Fig. 4b and S17†). The differences in wavelength could be due to the solvation of the anion radicals in PMMA (*ε*_r_ ∼ 5), which is less polar than DMF (*ε*_r_ ∼ 36). This result suggests that the injection of an electron into the diazaporphyrin core is coupled with intermolecular proton transfer.

**Fig. 6:**
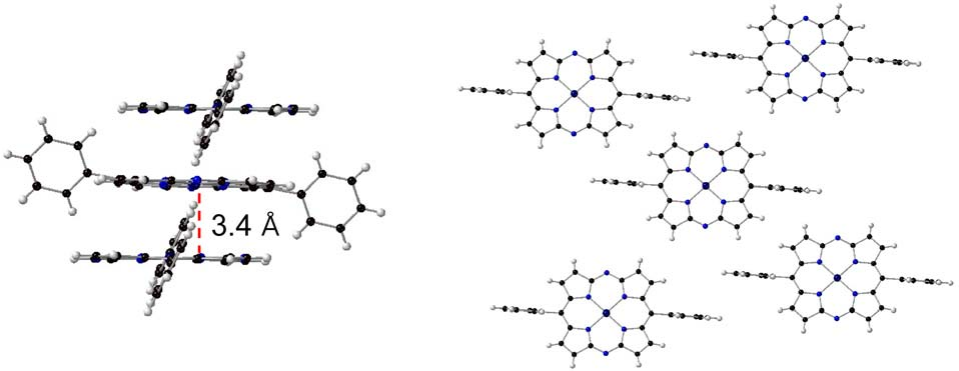
X-ray crystal structure of 4. Thermal ellipsoids are shown at the 50% probability level.

To investigate the influence of the hydrogen-bonding network, we evaluated the conductivity of 4, which forms a similar 1-D columnar packing structure in the crystal.^21^ In sharp contrast to 3-HOF, however, only weak CH–π interactions are present among the π-stacked columns in 4 (Fig. 6). The *φ*Σ*μ*_max_ of 4 (1.0 × 10^−8^ m^2^ V^−1^ s^−1^) is 20 times lower than that of 3-HOF (Fig. 5a). The calculated charge-transfer integrals of the LUMO of 3-HOF and 4 show no significant differences (Fig. S22†), indicating that it is likely that factors other than the molecular arrangement in the 1-D columnar stacking contribute to the electron mobility of 3-HOF.

**Fig. 7:**
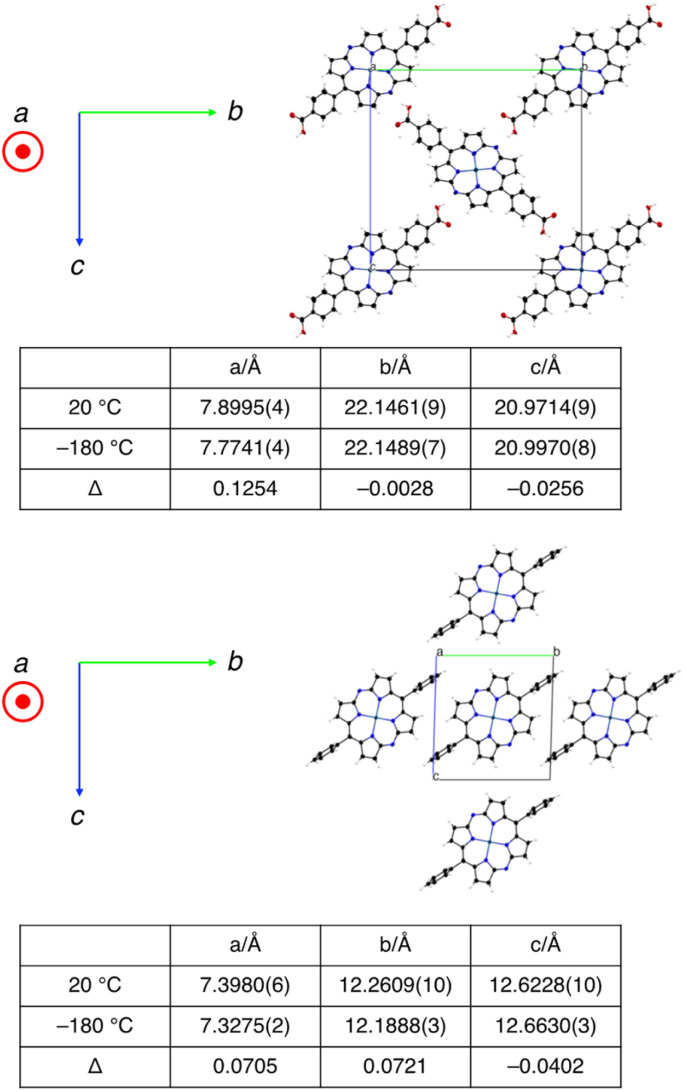
Crystal structures and dimensions of the crystal lattice at different temperatures for 3-HOF (top) and 4 (bottom).

We hypothesized that the suppression of molecular vibrations by the intermolecular hydrogen-bonding interactions is responsible for the effective charge mobility of 3-HOF.^22^ To examine their temperature-dependent structural changes, we conducted single-crystal X-ray diffraction analyses of 3-HOF and 4 at 20 and −180 °C. For this experiment, partially desolvated crystals of 3-HOF were used.^23^ The dimensions of the crystal lattices are summarized in Fig. 7. The thermal displacement of the lattice along the *a* axis, which is the direction of π–π stacking, is comparable for 3-HOF and 4. In contrast, the thermal displacement along the *b* and *c* axes is smaller for 3-HOF than for 4. The small structural deviation of 3-HOF with temperature originates from the stronger intermolecular interactions, predominantly due to the hydrogen-bonding networks. These results indicate that carrier mobility is influenced by not only molecular arrangement^25^ but also molecular vibration.^26^

An *I*–*V* measurement of a pressed pellet sample of 3-HOF was conducted by the two-probe method to evaluate its bulk conductivity (Fig. S18†). Surprisingly, 3-HOF shows distinct conductivity without any doping. The conductivity based on the *I*–*V* plot was 6.2 × 10^−9^ S cm^−1^, which is lower than other conductive PCMs (Table S2†). The EPR spectrum of 3-HOF in the solid state was recorded to confirm the carriers, revealing the existence of paramagnetic species with *g* value = 2.0482 (Fig. S20†). This implies the presence of unpaired electrons without doping. The electrochemical impedance measurement of 3-HOF confirmed its conductivity of 8.7–9.1 × 10^−7^ S cm^−1^ (Table S3†). Because the TRMC and impedance measurements suggested the high intrinsic charge mobility of 3-HOF, the resistance at the grain boundary of the pressed pellet sample could hinder long-range charge transport. Indeed, the activation energy derived from the impedance conductivity measurements in 3-HOF was estimated as 6.4 meV, which is consistent with the range of electron mobility in band-like conduction assessed by the FP-TRMC measurement.^20*d*^

## Conclusions

A Ni(ii) 5,15-diazaporphyrin functionalized with 4-carboxyphenyl groups at the *meso* positions afforded a HOF due to hydrogen-bonding interactions between the carboxy groups and *meso*-nitrogen atoms. FP-TRMC measurement elucidated that the diazaporphyrin-based HOF achieved a superior *φ*Σ*μ* value to the corresponding Ni(ii) 5,15-diphenyldiazaporphyrin. The intrinsic high n-type semiconductivity is likely due to the suppression of the molecular vibration by intermolecular hydrogen-bonding networks. These results should contribute to the development of conductive and stable porous materials.

## Data availability

All experimental and characterization data are available in the ESI.†

## Author contributions

T. S. carried out the synthesis and characterization and prepared the original draft. T. T. and H. I. evaluated the porosity of the HOF. Z. L., W. M. and Y. T. determined the conductivity of the HOF. S. S., R. M. and H. S. supervised the project and contributed to conceptualization, project administration and writing the manuscript.

## Conflicts of interest

There are no conflicts to declare.

## Supplementary Material

SC-015-D4SC03455D-s001

SC-015-D4SC03455D-s002
