# Supplementary material for: An n-type semiconducting diazaporphyrin-based hydrogen-bonded organic framework
Source: Chem Sci. 2024 Jul 11;15(32):12922–7. doi: 10.1039/d4sc03455d (PMC11323323; doi:10.1039/d4sc03455d)
Supplement: SC-015-D4SC03455D-s001 [file SC-015-D4SC03455D-s001.pdf]

Electronic Supplementary Material (ESI) for Chemical Science.
This journal is © The Royal Society of Chemistry 2024

Supporting Information

An n-Type Semiconducting Diazaporphyrin-Based Hydrogen-Bonded Organic Framework

Takahiro Sakurai, Tappei Tanabe, Hiroaki Iguchi, Zhuowei Li, Wakana Matsuda, Yusuke Tsutsui, Shu Seki,* Ryotaro Matsuda,* and Hiroshi Shinokubo*

Table of Contents

1. Instrumentation and materials 3

2. Experimental procedures and compound data 4

3. NMR spectra 7

4. Mass spectra 10

5. Crystal data 14

6. Powder X-ray diffraction analysis 16

7. Thermogravimetric analysis 17

8. Cyclic voltammograms 18

9. Photophysical properties 19

10. Electrical conductivity 21

11. Impedance spectroscopy 24

12. Transient absorption spectroscopy measurements 25

13. Electron paramagnetic resonance spectroscopy 26

14. DFT calculations 27

15. References 29

1. Instrumentation and materials

^{1}H NMR (500 MHz) and ^{13}C NMR (126 MHz) spectra were recorded on a Bruker AVANCE III HD spectrometer. Chemical shifts were reported as the delta scale in ppm relative to $CHCl_3$ (δ = 7.26 ppm) and DMSO (δ = 2.50 ppm) for ^{1}H NMR and $CDCl_3$ (δ = 77.16 ppm) and DMSO (δ = 39.52 ppm) for ^{13}C NMR. UV/vis/NIR absorption spectra were recorded on a JASCP V 670 spectrometer. Fourier transform infrared (FT-IR) spectra were recorded on a Shimadzu IR Sprint-T using ATR. High-resolution atmospheric pressure chemical ionization time-of-flight (APCI-TOF) mass spectra were taken on a Bruker micrOTOF instrument using a positive ionization mode. Single crystal X-ray data were obtained using a Rigaku CCD diffractometer (Saturn 724 with MicroMax-007) with Varimax Mo optics. Powder X-ray diffraction (PXRD) patterns at room temperature were measured on a Rigaku MiniFlex600 operating at 40 kV/15 mA producing Cu Kα radiation (λ = 1.5406 Å) at room temperature ranging from 3° to 55° except for any additional information. The adsorption isotherm measurements for N_2 and CO_2 were performed using an automatic volumetric adsorption apparatus (BELSORP-max equipped with a cryostatic temperature controller; MicrotracBEL Corp.). The TGA profile was recorded using STA 2500 Regulus (Netzsch Japan Corp.). Cyclic voltammograms were obtained under the following conditions. Solvent: DMF, electrolyte: 0.1 M Bu_4NPF_6, working electrode: glassy carbon, counter electrode: Pt, reference electrode: $Ag/AgNO_3$, scan rate: 0.05 V/s, reference redox couple: Fc/Fc^{+}. All potentials are referenced to the potential of a ferrocene/ferrocenium cation couple.

N,N-Dimethylformamide (DMF) was purchased from Wako Pure Chemical Industries, Ltd. as a dehydrated grade. Diphenyldiazaporphyrin **4** was prepared according to the literature.[1] Unless otherwise noted, materials obtained from commercial suppliers were used without further purification.

2. Experimental procedures and compound data

Synthesis of free-base 10,20-di(*p*-carboxymethylphenyl)-5,15-diazaporphyrin 1

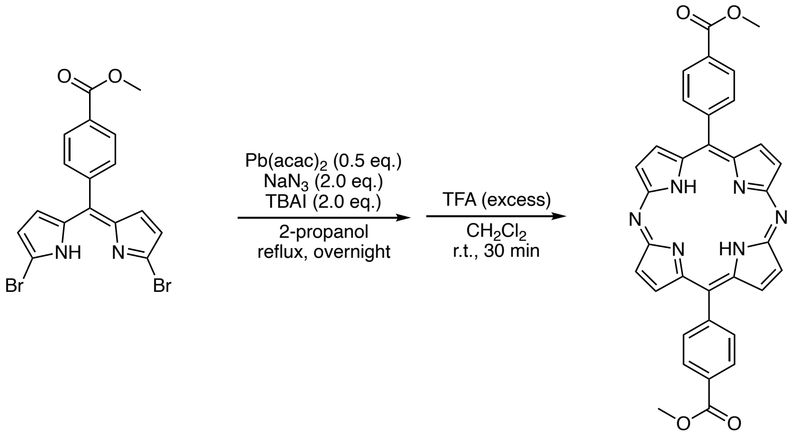

1

Diazaporphyrin **1** was synthesized according to the literature with some modifications.[2] In a flask, *meso*(*p*-carboxymethylphenyl)dibromodipyrrin (2.62 g, 6.00 mmol), $Pb(acac)_2$ (1.22 g, 3.00 mmol), NaN_3 (0.78 g, 12 mmol), tetrabutylammonium iodide (4.40 g, 12 mmol), and 2-propanol (600 mL) were added. The reaction mixture was refluxed overnight. After cooling to room temperature, water (100 mL) was added. The reaction mixture was evaporated until the amount of solvent became c.a. 200 mL. The residue was filtered and dissolved in CH_2Cl_2 (150 mL). Trifluoroacetic acid (2 mL, 26 mmol) was added to the solution and stirred at room temperature for 30 min. Then, saturated $NaHCO_3$ aq. (100 mL) was added. The reaction mixture was extracted by CH_2Cl_2. The organic layer was dried over anhydrous Na_2SO_4 and concentrated in *vacuo*. The residue was purified by column chromatography (SiO_2) with CH_2Cl_2/ethyl acetate = 10/1. The obtained solid was washed with acetone to obtain **1** (545 mg, 16 %) as a purple solid. ^{1}H NMR (500 MHz, $CDCl_3$, 298 K): δ = 9.33 (d, J = 5.0 Hz, 4H), 8.98 (d, J = 5.0 Hz, 4H), 8.51 (d, J = 8.0 Hz, 4H), 8.29 (d, J = 8.0 Hz, 4H), 4.15 (s, 6H), –2.82 (s, 2H) ppm; ^{13}C NMR (126 MHz, $CDCl_3$, 298 K): δ =167.2, 144.1, 134.9, 133.6, 133.3, 130.5, 128.6, 121.6, 52.7 ppm; Two of aromatic ^{13}C NMR signals were missing possibly because of overlapping. HRMS (APCI): $[M+H]^+$ Calcd. for $C_{34}H_{25}N_6O_4$ 581.1932; Found 581.1934.

Synthesis of nickel(II) 10,20-di(*p*-carboxymethylphenyl)-5,15-diazaporphyrin 2

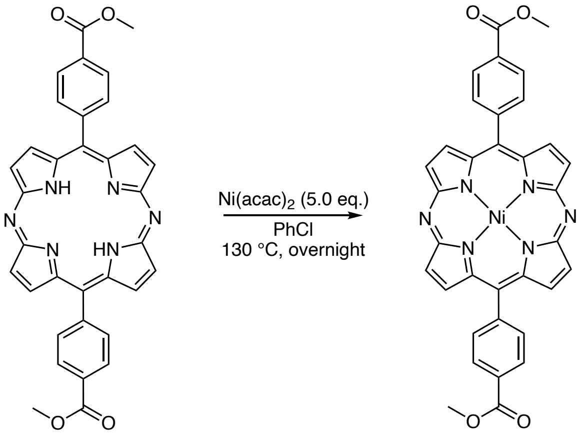

In a flask, **1** (545 mg, 0.94 mmol), Ni(acac)$_2$ (1.20 g, 4.7 mmol), and chlorobenzene (35 mL) were added. The reaction mixture was stirred at 130 °C for overnight. After cooling to room temperature, the reaction mixture was subjected to silica gel and eluted with hexane until chlorobenzene disappeared. Then, the desired Ni complex was eluted by CH_2Cl_2/ethyl acetate = 10/1. Recrystallization from CH_2Cl_2/MeOH afforded **2** (517 mg, 86%) as a purple solid. ^{1}H NMR (500 MHz, $CDCl_3$, 298 K): δ = 9.06 (d, J = 5.0 Hz, 4H), 8.76 (d, J = 5.0 Hz, 4H), 8.47 (d, J = 8.0 Hz, 4H), 8.16 (d, J = 8.0 Hz, 4H), 4.14 (s, 6H) ppm; ^{13}C NMR (126 MHz, $CDCl_3$, 298 K): δ = 167.2, 150.3, 143.7, 143.5, 134.3, 134.2, 134.0, 130.4, 128.5, 121.1, 52.7 ppm; HRMS (APCI): $[M+H]^+$ Calcd. for $C_{34}H_{22}N_6O_4Ni$ 637.1129; Found 637.1105.

Synthesis of nickel(II) 10,20-di(*p*-carboxyphenyl)-5,15-diazaporphyrin 3

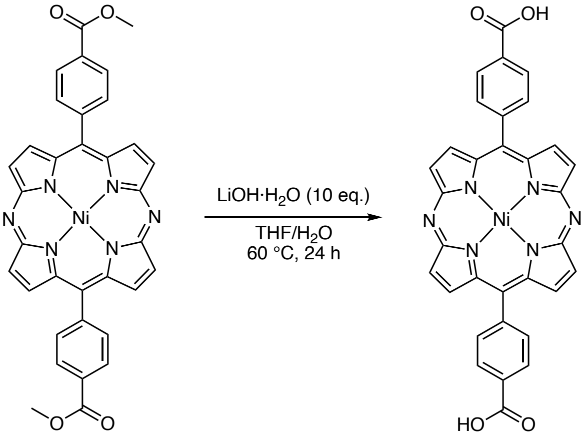

In a flask, **2** (257 mg, 0.40 mmol), LiOH·H_2O (169 mg, 4.0 mmol), and THF/H_2O =15/15 mL were added. The reaction mixture was stirred at 60 °C for 24 h. After cooling to room temperature, the reaction mixture was evaporated to remove THF. Then, 2 M HCl aq. was added dropwise at 0 °C until the pH of the solution became 3. The precipitate was collected by filtration and washed

with MeOH to afford **3** (232 mg, 95%) as a purple solid. ^{1}H NMR (500 MHz, DMSO-d_6, 298 K): δ = 13.38 (br, 2H), 8.97 (br, 4H), 8.61 (br, 4H), 8.41 (d, J = 6.4 Hz, 4H), 8.19 (d, J = 6.4 Hz, 4H) ppm; ^{13}C NMR (126 MHz, DMSO-d_6, 298 K): δ = 167.4, 149.0, 142.4, 142.3, 134.2, 133.9, 133.6, 130.8, 128.2, 120.4 ppm; HRMS (APCI): $[M+H]^+$ Calcd. for $C_{32}H_{18}N_6O_4Ni$ 609.0816; Found 609.0828.

Synthesis of 3-3-HOF

The single crystal suitable for X-ray analysis of **3-3-HOF** was prepared by vapor diffusion method using NMP as a good solvent and MeCN as a poor solvent. The powdery sample was synthesized using the liquid–liquid diffusion method using the same solvent as the vapor diffusion method.

3. NMR spectra

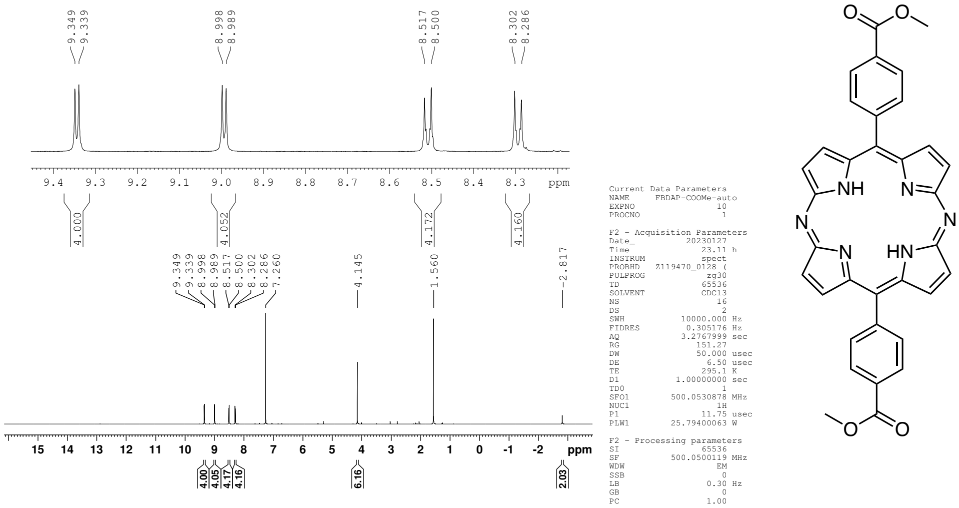

Figure S1. ^{1}H NMR spectrum of **1** in $CDCl_3$ at 25 °C.

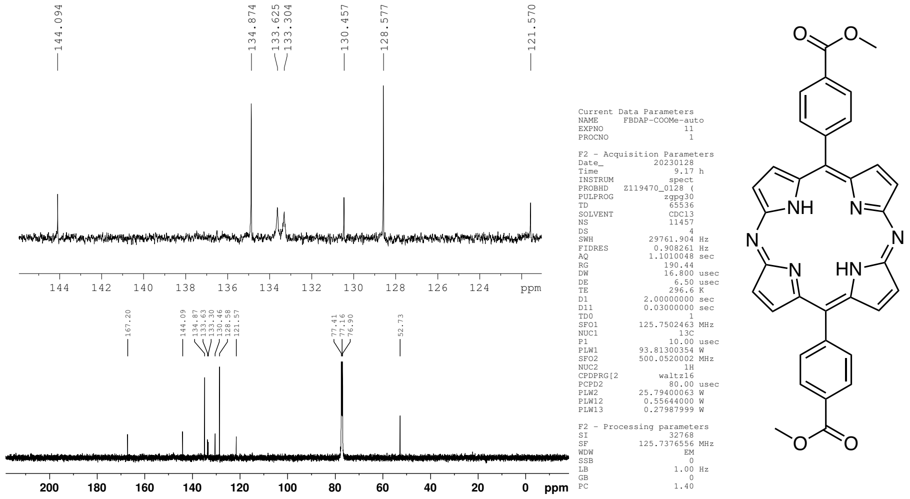

Figure S2. ^{13}C NMR spectrum of **1** in $CDCl_3$ at 25 °C.

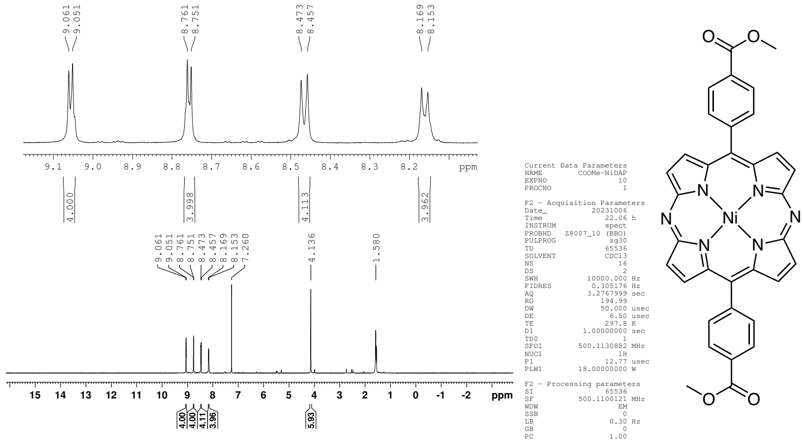

Figure S3. ^{1}H NMR spectrum of **2** in $CDCl_3$ at 25 °C.

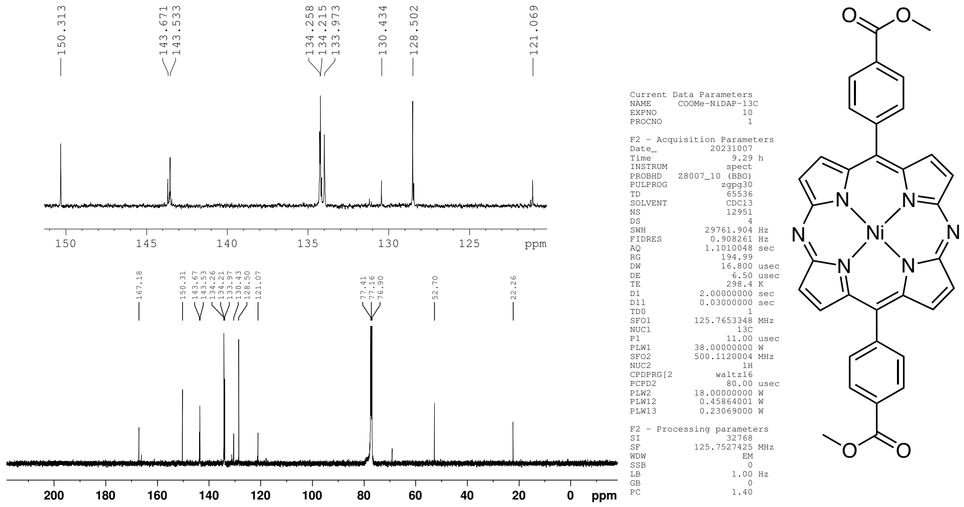

Figure S4. ^{13}C NMR spectrum of **2** in $CDCl_3$ at 25 °C.

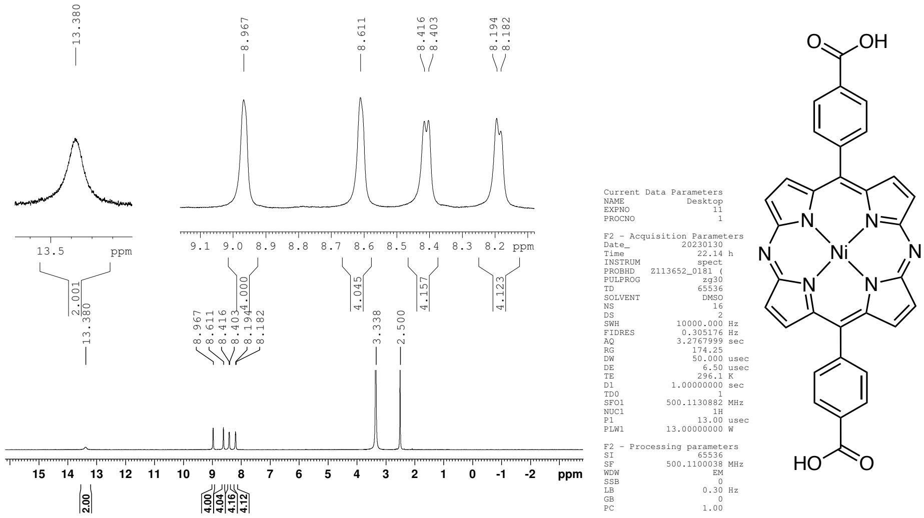

Figure S5. ^{1}H NMR spectrum of **3** in DMSO-d_6 at 25 °C.

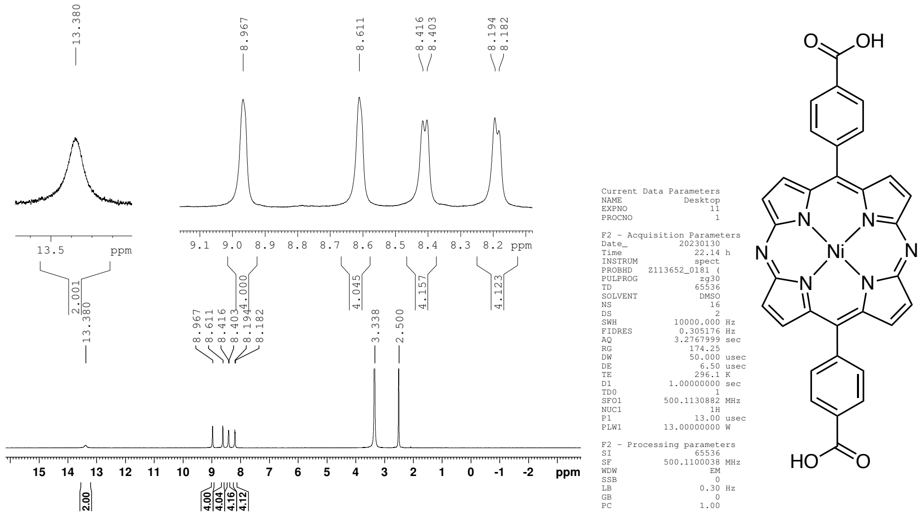

Figure S6. ^{13}C NMR spectrum of **3** in DMSO-d_6 at 25 °C.

4. Mass spectra

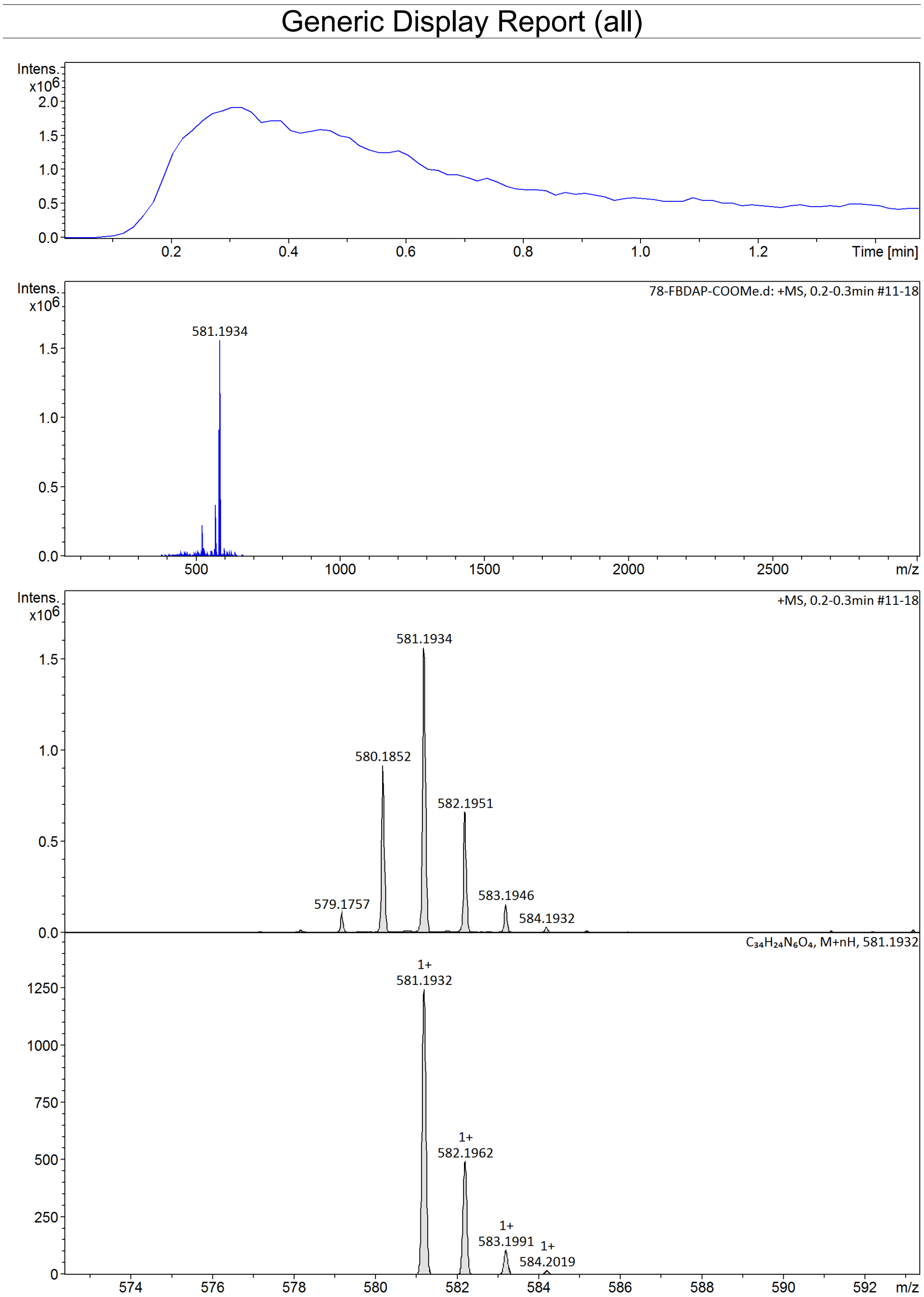

Figure S7. APCI-TOF-MS spectrum of **1**.

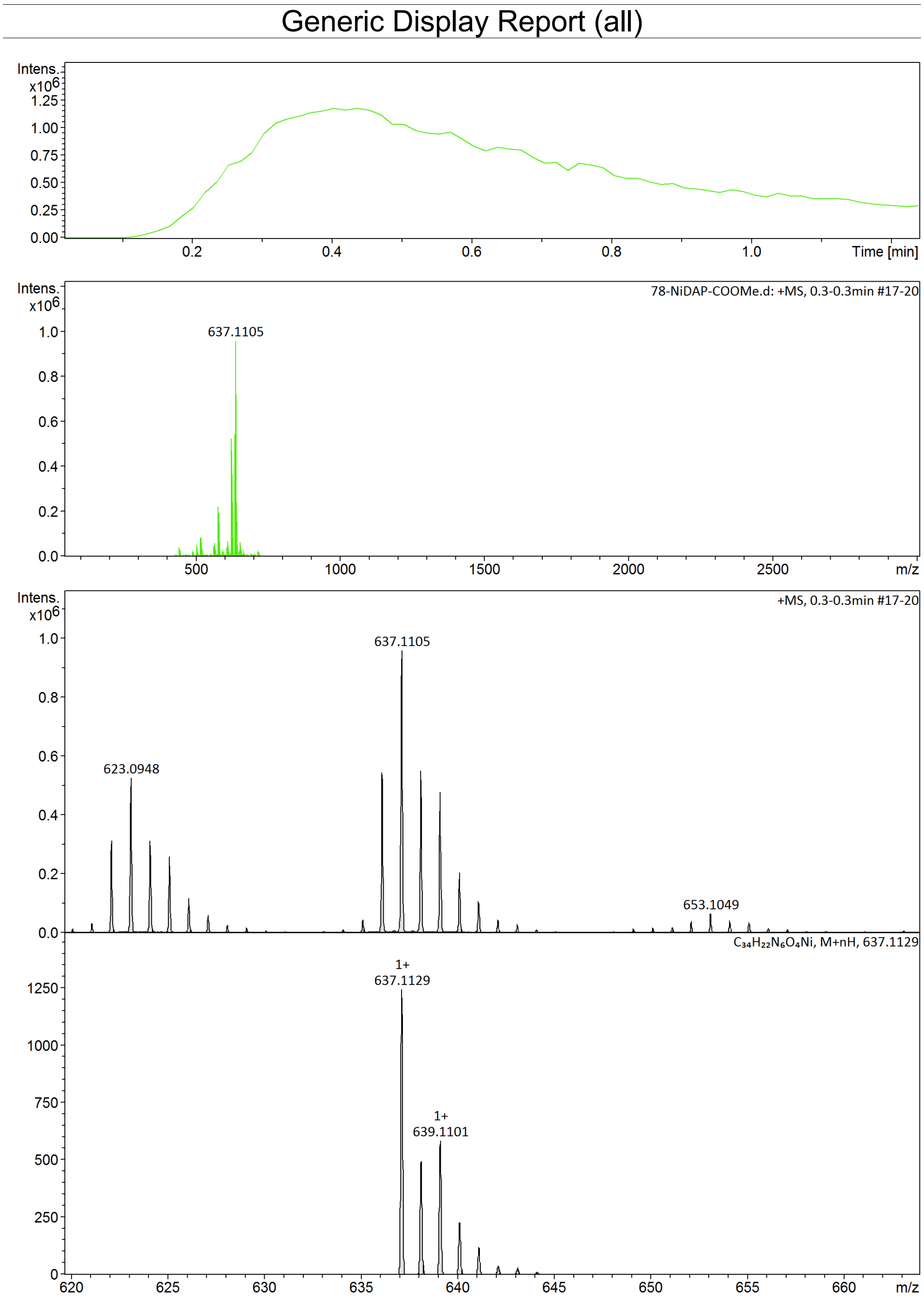

Figure S8. APCI-TOF-MS spectrum of **2**.

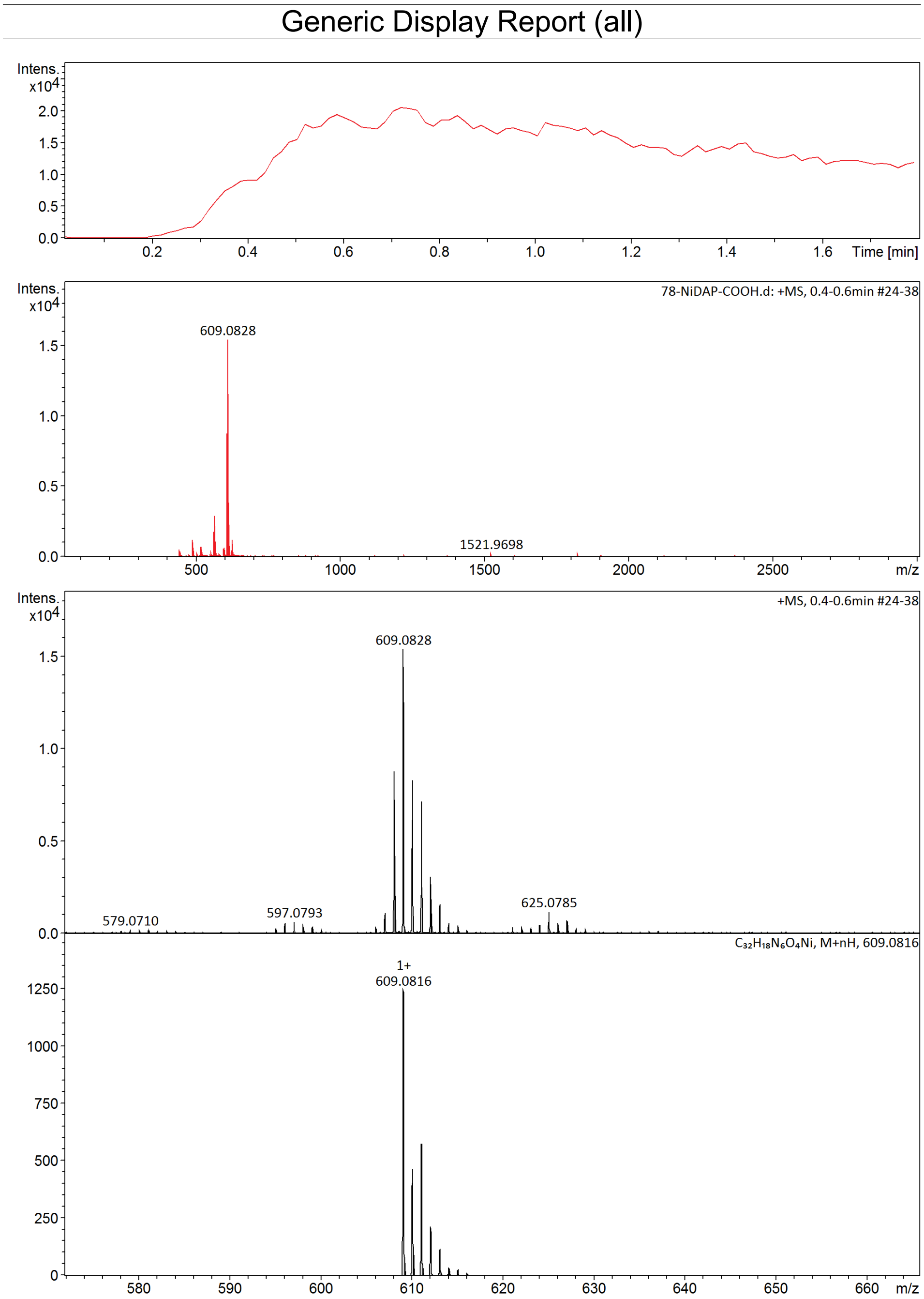

Figure S9. APCI-TOF-MS spectrum of **3**.

5. FT-IR Spectrum

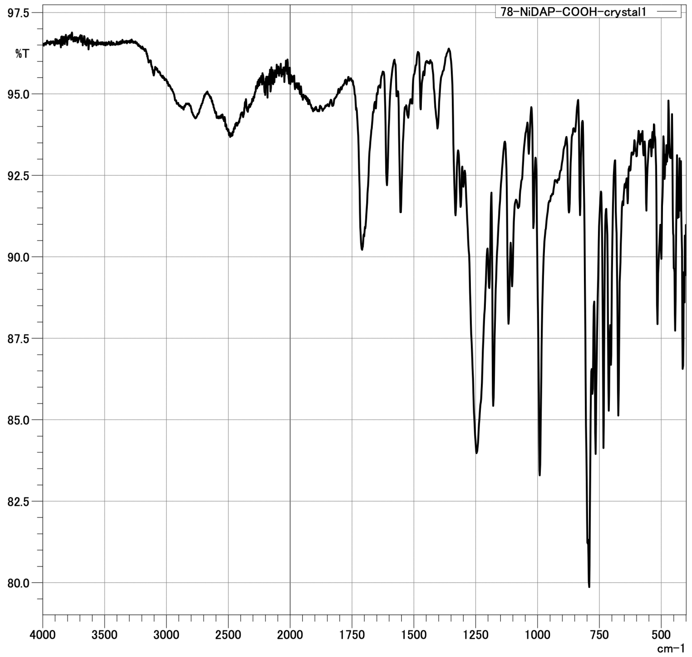

Figure S10. FT-IR spectrum of a powder sample of **3-HOF**.

Crystal data

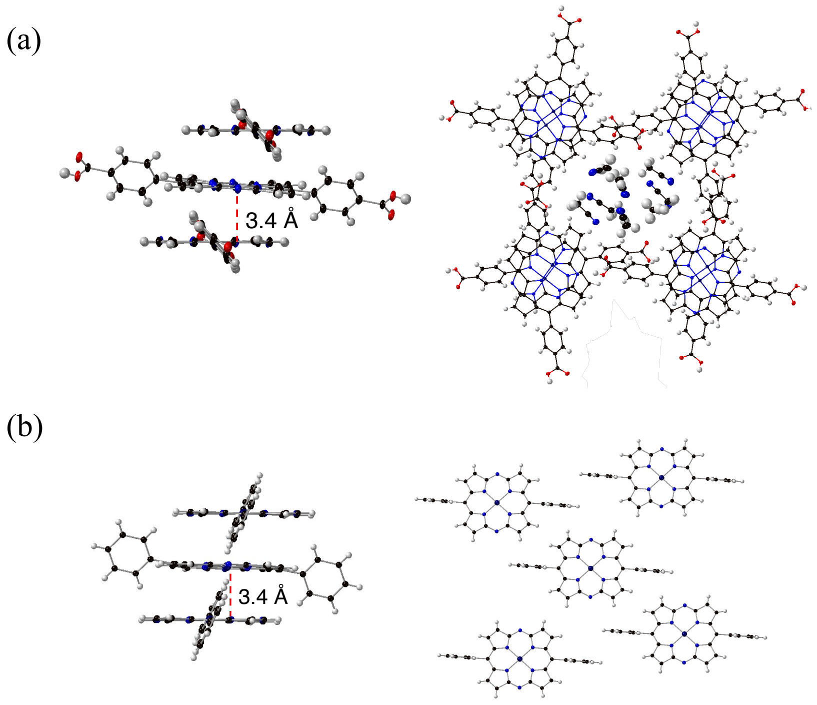

Figure S11. X-ray crystal structures of (a) **3-HOF** and (b) **4**. Thermal ellipsoids are shown at the 50% probability level.

Table S1. Crystallographic data of **3-HOF**.

compound	**3-HOF**
Formula	$C_{32}H_{18}N_6NiO_4$ $4(C_2H_3N)$
Formula weight	773.45
Crystal system	monoclinic
Space group	$P2_1/n$ (No. 14)
Crystal color	purple
Crystal description	plate
a [Å]	7.6006(2)
b [Å]	22.3585(4)
c [Å]	20.9221(4)
β [°]	90.948(2)
V [Å^3]	3554.97(13)
Z	4
d_{calcd} [g cm^{-3}]	1.445
R_1 ($I > 2\sigma(I)$)	0.0741
wR_2 (all data)	0.1761
Goodness-of-fit	1.073
Temperature [K]	93(2)
Solvent	NMP/MeCN
CCDC No.	2331973

6. Powder X-ray diffraction analysis

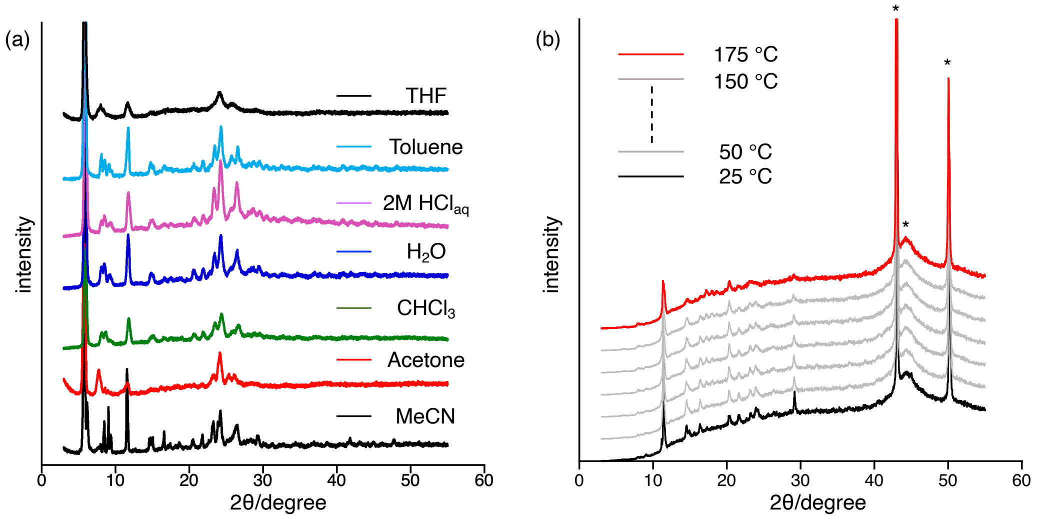

Figure S12. PXRD patterns of **3-HOF** (a) after soaking various solvents for 12 h and (b) at various temperatures. Peaks with an asterisk (*) originated from the base.

7. Thermogravimetric analysis

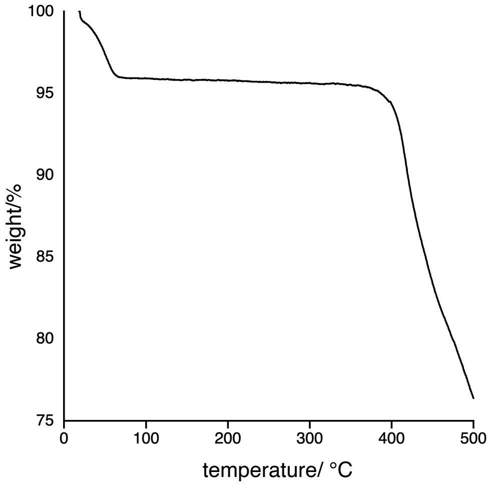

Figure S13. TGA profile of **3-HOF**. The heating gradient was 5.0 °C min^{-1}. The weight ratio of acetonitrile calculated from the crystal structure was not matched with this result, probably because some amount of acetonitrile was desolvated during the drying process.

8. Cyclic voltammograms

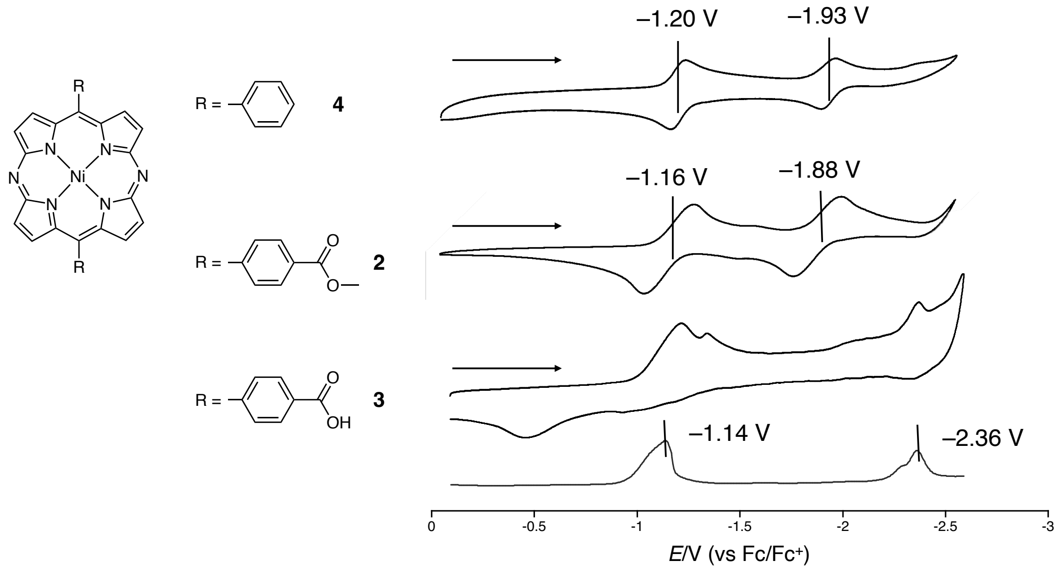

Figure S14. Cyclic voltammograms of **4**, **2**, and **3**.

9. Photophysical properties

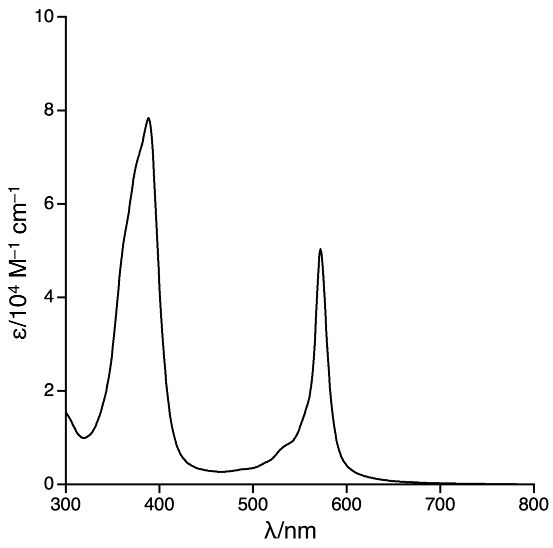

Figure S15. UV-vis absorption spectrum of **3** in DMF.

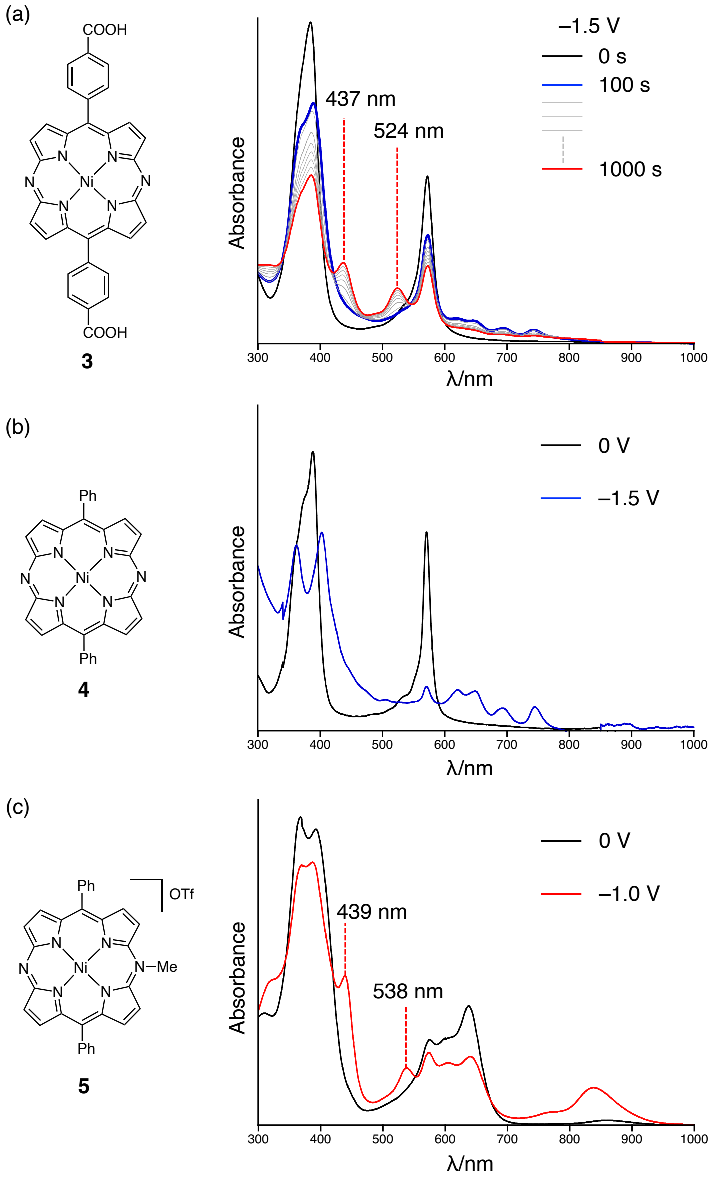

Figure S16. Change of absorption spectra of (a) **3**, (b) **4**, and (c) **5** in DMF upon one-electron reduction.

10. Electrical conductivity

Time Resolved Microwave Conductivity (TRMC) and Transient Absorption Spectroscopy(TAS) Transient photoconductivity was measured by a Flush Photolysis-TRMC setup. A resonant cavity with Q ~ 2500 was used to obtain a high degree of sensitivity in the conductivity measurement. Proving microwave frequency and power were set at ~9.1 GHz and 3.0 mW, respectively, such that the electric field of the microwave was sufficiently small not to disturb the translational motion of charge carriers. The observed value of photoconductivity converted to the product of the quantum yield ϕ and the sum of charge-carrier mobilities $\sum\mu$ by $\phi\sum\mu=\Delta\sigma/eI_0F_{\mathrm{light}}$, where e, I_0, F_{light}, and $\Delta\sigma$ are elementary charge, incident photon density of excitation laser (photons per m^2), a correction factor (m^{-1}) and a transient photoconductivity, respectively. The sample was set at the point of electric field maximum in a resonant cavity. FP-TRMC experiments were performed at room temperature and under Ar-saturated conditions by continuous flowing for > 10 min. The measurements of all the samples were performed for polycrystalline samples. These samples were fixed on a quartz substrate by poly(methymethacrylate) binders.

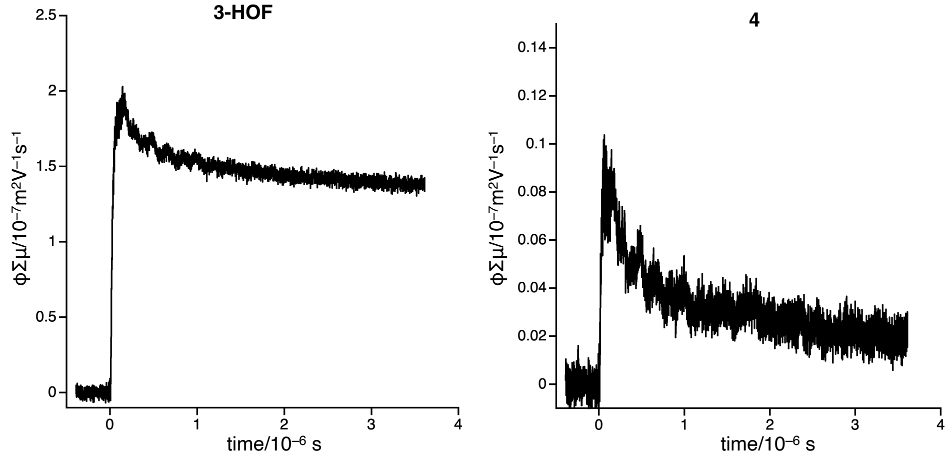

Figure S17. FP-TRMC measurements of **3-HOF**(left) and **4**(right) in polycrystalline form.

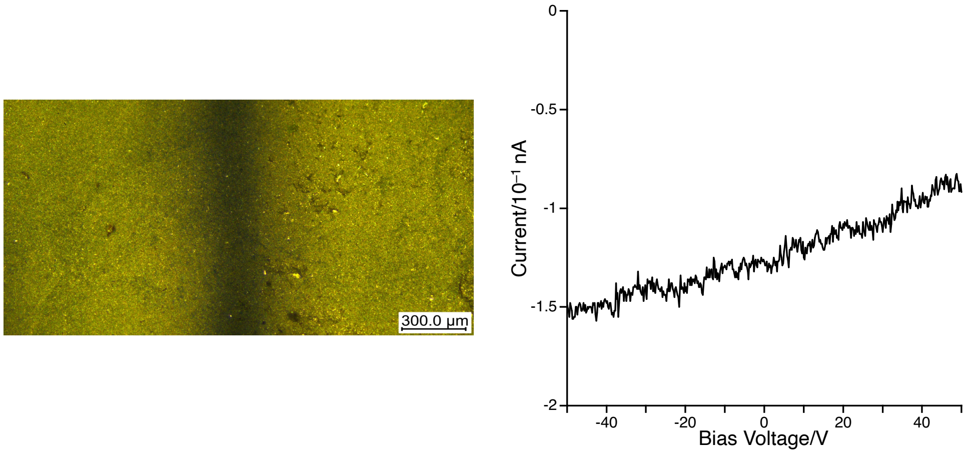

Figure S18. Optical microscope image of measured sample (left), and *I–V* plot for pressed pellet of **3-HOF** (right).

Table S2. Selected conduction properties of PCMs

	Material	Carrier	$\varphi\Sigma\mu$ /$m^2V^{-1}s^{-1}$	σ/Scm^{-1}	σ method	Ref.
	**3-HOF**	electron	2.0×10^{-7}	6.2×10^{-9}	2-probe pellet	This work
Porphyrin-based PCM	COF-66	hole	1.7×10^{-9}			6
	COF-366	hole	4.1×10^{-9}			6
	H_2P-COF	hole	1.8×10^{-8}			7
	CuP-COF	ambipolar	9.0×10^{-9}			7
	ZnP-COF	electron	1.2×10^{-8}			7
	Pd-porphyrin Zn-SURMOF2	hole	1.2×10^{-8}			8
	Free base porphyrin Zn-SURMOF2	hole	$1.5–2.0 \times 10^{-8}$			8
TTF-based HOFs[a]	HOF-110	hole	–	2.2×10^{-8}	2-probe pellet	9
	HOF-110@I_2-1	hole	–	2.7×10^{-7}	2-probe pellet	9
	HOF-110@I_2-2	hole	–	6.0×10^{-7}	2-probe pellet	9
	MUV-20a	hole	–	6.1×10^{-7}	2-probe pellet	10
	MUV-20b	hole	–	1.4×10^{-6}	2-probe pellet	10
	MUV-21	hole	-	6.2×10^{-9}	2-probe pellet	10

TTF-based PCM	Zn_2TTFTB	hole	3×10^{-9}	5.0×10^{-4}	time-resolved terahertz spectroscopy	φΣμ: 11<br>σ: 12
	TTF-Ph-COF	–	1.1×10^{-9}	10^{-5}	2-probe pellet	13
	TTF-Py-COF	–	5.0×10^{-10}	10^{-6}	2-probe pellet	13
Others	$Fe_2(BDP)_3$	hole	$\sim 10^{-8}$	3.5×10^{-7}	2-probe single crystal	14
	$K_{0.98}Fe_2(BDP)_3$	hole	$\sim 10^{-9}$	2.8×10^{-2}	2-probe single crystal	14
Quinone-based PCM	$[Mn_2(Cl_2dhbq)_3][(Me_4N)_2]$		–	1.1×10^{-13}	2-probe pellet	15
	$[Mn_2(Cl_2dhbq)_3][(Me_4N)_2]$@$Na[C_{10}H_8]$	electron	–	2.3×10^{-8}	2-probe pellet	15
	$[Fe_2(Cl_2dhbq)_3][(Me_2NH_2)_2]$ (as synthesized)	electron		1.4×10^{-2}	2-probe pellet	16
	$[Fe_2(Cl_2dhbq)_3][(Me_2NH_2)_2]$ (activated)	electron		1.0×10^{-3}	2-probe pellet	16
NDI-based PCM[b]	$Cu(DPNDI)_2$	electron		1.0×10^{-5}	2-probe single crystal	17
	Cd(DPNDI) (as synthesized)	electron		7.6×10^{-6}	2-probe pellet	18
	Cd(DPNDI) (activated)	electron		3.7×10^{-2}	2-probe pellet	18

a; TTF = tetrathiafulvalene, b; NDI = naphthalenediimide

11. Impedance spectroscopy

Static and electrochemical conductivity measurements were carried out with ALS760E two-probe AC impedance measurement set-up. The powdered samples were compressed to ~ 0.5 mm in thickness and 5 mm in diameter. The HOF pellets were placed between two electrodes in an environmental chamber. All of the temperatures were kept for at least one hour so that the sample would achieve the equilibrium. Electrical conductivity was measured by the equation: $\sigma = L/(R \cdot A)$, where σ, L, R, and A are the conductivity (S cm^{-1}), thickness of the pellet (cm), resistance of the pellet (Ω), and area of the pellet (cm^2), respectively.

Table S3. Electrochemical impedance measurement of **3-HOF** at different temperatures.

°C	σ/Scm^{-1}, [a]
25	8.7×10^{-7}
35	9.0×10^{-7}
45	8.9×10^{-7}
55	9.0×10^{-7}
65	9.1×10^{-7}

[a] Temperature dependence of the conductivity derived from the impedance measurements was analysed by Arrhenius equation as:

$$\sigma = \sigma_0 \exp\left(-\frac{E_\mathrm{a}}{kT}\right),$$

where E_a, σ_0, k, and T are activation energy, a preexponential factor conductivity, Boltzmann constant, and temperature, respectively. The value of E_a was estimated as $E_\mathrm{a} =$ 6.4 meV.

12. Transient absorption spectroscopy measurements

Transient absorption spectrum was carried out by the following setup. Identical polycrystalline sample to TRMC measurements was employed in the spectroscopy. The sample was photo-excited using third harmonics of nanosecond Nd:YAG pulsed laser (355 nm, 1.0 mJ, 10 Hz, EKSPLA NT341A). Continuous white light from a 150 W Xenon lamp (L14972-01) was used as an probe source and transmitted light has been spectrally dispersed by a monochromator with focal plane of 300 mm (C11119-04, Hamamatsu Photonics). It was also dispersed in the time domain with a high dynamic range streak camera (C13410-01A, Hamamatsu Photonics) and detected with a digital CMOS camera (ORCA-Flash4.0 V3 C13440-20CU, Hamatsu Photonics). All the electrical timing was adjusted with a digital delay generator (DG645, Stanford Research Systems).

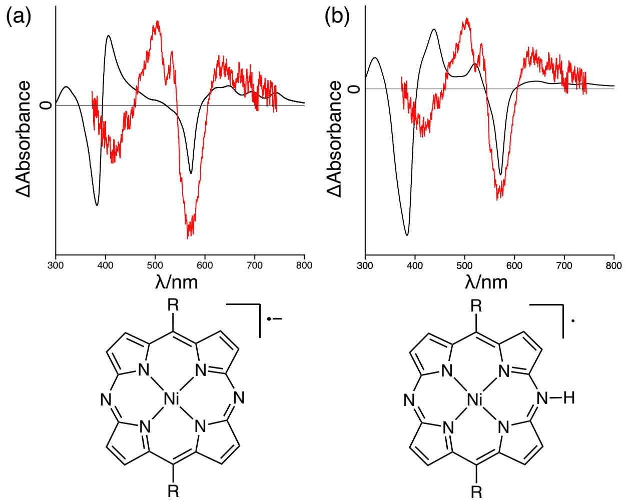

Figure S19. Comparison of the transient absorption spectrum of **3-HOF** in a PMMA film (red line) with differential absorption spectra between **3** and its one-electron reduced species in DMF (black line). (a) After 100 s and (b) after 1000 s.

13. Electron paramagnetic resonance spectroscopy

A JEOL JES-FA200 X-band spectrometer was used to obtain electron paramagnetic resonance (EPR) spectra at room temperature and under ambient conditions. In the EPR spectrum, **3-HOF** exhibits a signal at $g = 2.0482$.

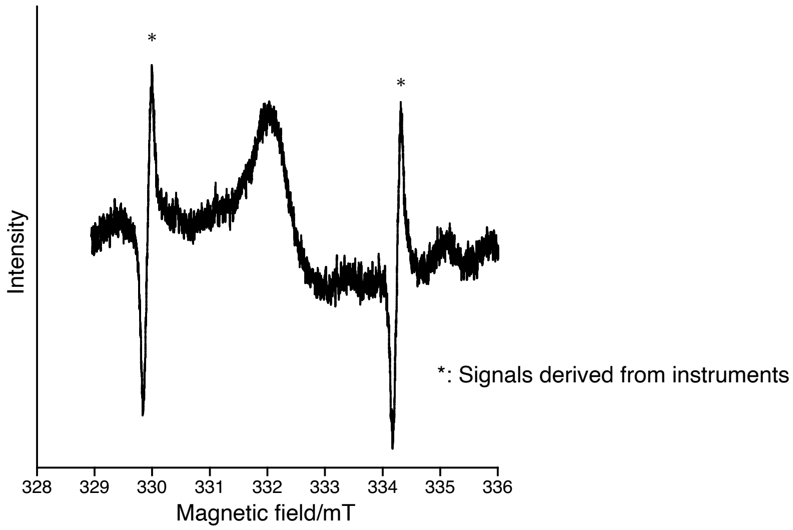

Figure S20. EPR spectrum of **3-HOF** in the solid state.

14. DFT calculations

All calculations were carried out using the Gaussian 09 program.[3] Initial geometries of **3** and **4** are obtained from their X-ray crystal structures. The structures of **3** and **4** were fully optimized without any symmetry restriction at Becke's three-parameter hybrid exchange functional and the Lee–Yang–Parr correlation functional (B3LYP)[4] with a basis set consisting of SDD[5] for Ni and 6-31G(d,p) for the rest. The calculations of transfer integrals were carried out with no optimization, and at PW91VWN/TZVP+SDD level.

(a)

(b)

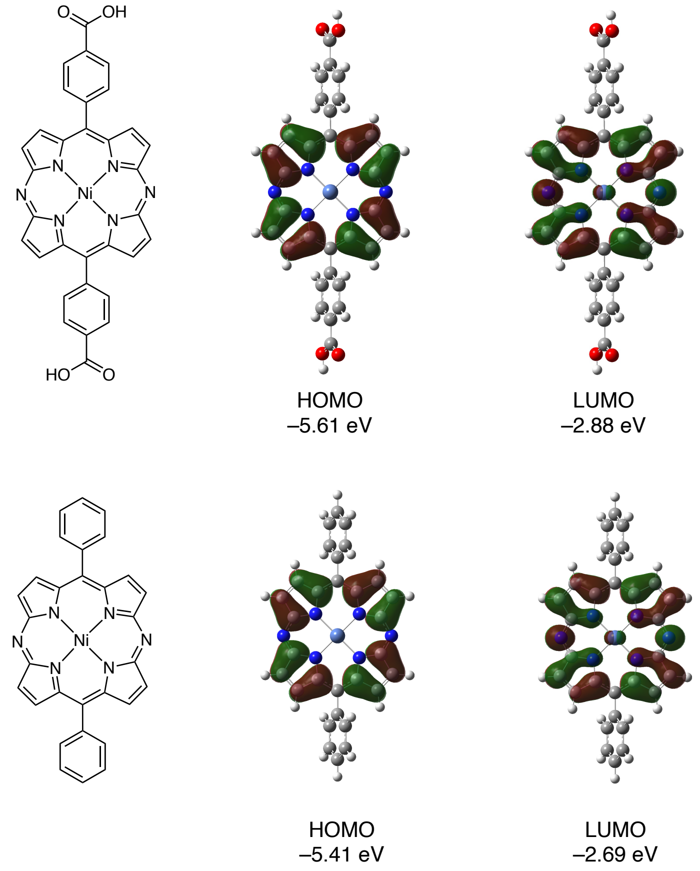

Figure S21. Calculated molecular orbitals of **3** and **4**.

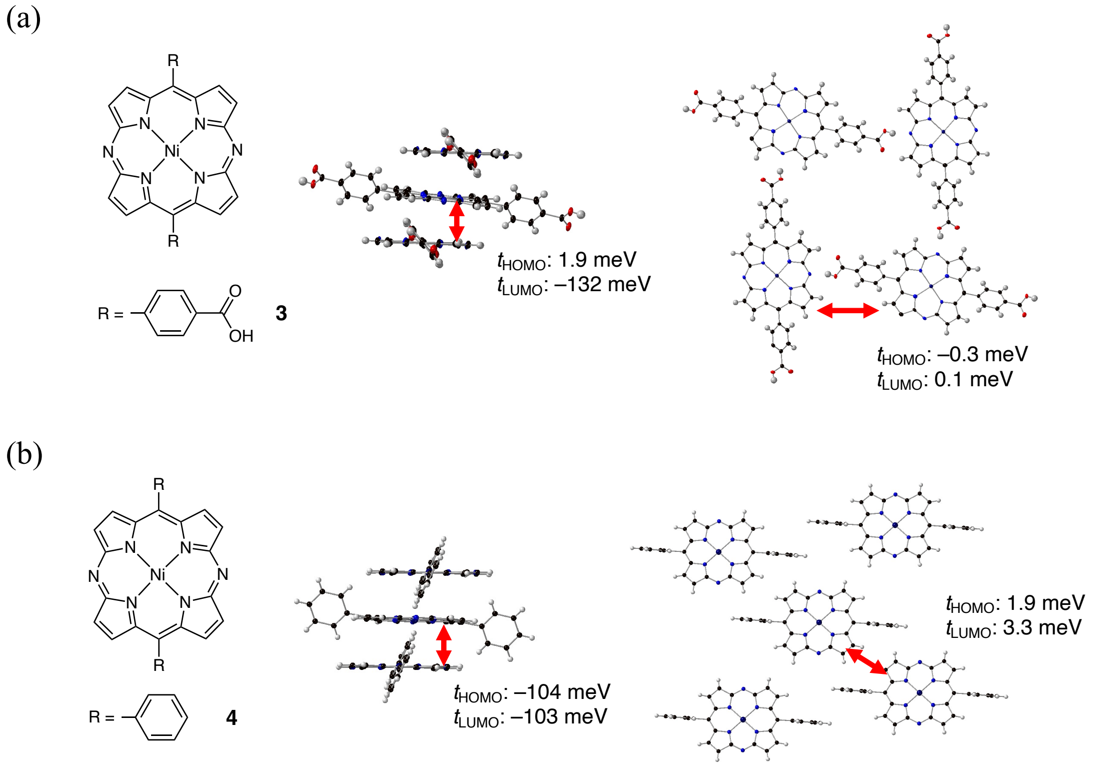

Figure S22. Calculated transfer integrals of (a) **3-HOF** and (b) **4**.

15. References

1. S. Mori, T. Sakurai, T. Nishimura, N. Fukui, Y. Miyake, H. Shinokubo, *J. Porphyrins Phthalocyanines* **2023**, A-G
2. Y. Matano, T. Shibano, H. Nakano, Y. Kimura, H. Imahori, *Inorg. Chem.* **2012**, *51*, 12879–12890.
3. Gaussian 09, Revision D.01, Frisch, M. J.; Trucks, G. W.; Schlegel, H. B.; Scuseria, G. E.; Robb, M. A.; Cheeseman, J. R.; Scalmani, G.; Barone, V.; Mennucci, B.; Petersson, G. A.; Nakatsuji, H.; Caricato, M.; Li, X.; Hratchian, H. P.; Izmaylov, A. F.; Bloino, J.; Zheng, G.; Sonnenberg, J. L.; Hada, M.; Ehara, M.; Toyota, K.; Fukuda, R.; Hasegawa, J.; Ishida, M.; Nakajima, T.; Honda, Y.; Kitao, O.; Nakai, H.; Vreven, T.; Montgomery, Jr., J. A.; Peralta, J. E.; Ogliaro, F.; Bearpark, M.; Heyd, J. J.; Brothers, E.; Kudin, K. N.; Staroverov, V. N.; Kobayashi, R.; Normand, J.; Raghavachari, K.; Rendell, A.; Burant, J. C.; Iyengar, S. S.; Tomasi, J.; Cossi, M.; Rega, N.; Millam, J. M.; Klene, M.; Knox, J. E.; Cross, J. B.; Bakken, V.; Adamo, C.; Jaramillo, J.; Gomperts, R.; Stratmann, R. E.; Yazyev, O.; Austin, A. J.; Cammi, R.; Pomelli, C.; Ochterski, J. W.; Martin, R. L.; Morokuma, K.; Zakrzewski, V. G.; Voth, G. A.; Salvador, P.; Dannenberg, J. J.; Dapprich, S.; Daniels, A. D.; Farkas, Ö.; Foresman, J. B.; Ortiz, J. V.; Cioslowski, J.; Fox, D. J. Gaussian, Inc., Wallingford CT, 2009.
4. (a) Becke, A. D. *Phys. Rev. A* **1988**, *38*, 3098. (b) Lee, C.; Yang, W.; Parr, R. G. *Phys. Rev. B* **1988**, *37*, 785.
5. Dolg, M.; Wedig, U.; Stoll, H.; Preuss, H. *J. Chem. Phys.* **1987**, *86*, 866.
